# prPred: A Predictor to Identify Plant Resistance Proteins by Incorporating k-Spaced Amino Acid (Group) Pairs

**DOI:** 10.3389/fbioe.2020.645520

**Published:** 2021-01-21

**Authors:** Yansu Wang, Pingping Wang, Yingjie Guo, Shan Huang, Yu Chen, Lei Xu

**Affiliations:** ^1^School of Electronic and Communication Engineering, Shenzhen Polytechnic, Shenzhen, China; ^2^School of Life Science and Technology, Harbin Institute of Technology, Harbin, China; ^3^Department of Neurology, The 2nd Affiliated Hospital of Harbin Medical University, Harbin, China; ^4^College of Information and Computer Engineering, Northeast Forestry University, Harbin, China

**Keywords:** prPred, plant R protein, CKSAAP, CKSAAGP, support vector machine

## Abstract

To infect plants successfully, pathogens adopt various strategies to overcome their physical and chemical barriers and interfere with the plant immune system. Plants deploy a large number of resistance (R) proteins to detect invading pathogens. The R proteins are encoded by resistance genes that contain cell surface-localized receptors and intracellular receptors. In this study, a new plant R protein predictor called prPred was developed based on a support vector machine (SVM), which can accurately distinguish plant R proteins from other proteins. Experimental results showed that the accuracy, precision, sensitivity, specificity, F1-score, MCC, and AUC of prPred were 0.935, 1.000, 0.806, 1.000, 0.893, 0.857, and 0.948, respectively, on an independent test set. Moreover, the predictor integrated the HMMscan search tool and Phobius to identify protein domain families and transmembrane protein regions to differentiate subclasses of R proteins. prPred is available at https://github.com/Wangys-prog/prPred. The tool requires a valid Python installation and is run from the command line.

## Introduction

Plant pathogens can disturb the plant immune system to support their growth and development within plant tissue. The propagation and spread of pathogens threaten food security and cause crop and economic losses. To recognize invading pathogens, plants have evolved various disease resistance proteins (R proteins). There are two main categories of plant R proteins: membrane-bound pattern recognition receptors (PRRs) and intracellular resistance receptors. PRRs are comprised of two receptor classes, receptor-like proteins (RLPs) and receptor-like kinases (RLKs), that are located on the plant plasma membrane as the first layer of the surveillance system to detect microbe-derived molecular patterns. PRRs typically contain highly variable extracellular domains, such as lysin motif (LysM), leucine-rich repeat (LRR), and lectin domains (Zhou and Yang, 2016). The majority of intracellular resistance receptors (NBS-LRRs or NLRs) are nucleotide-binding sites (NBSs) and LRR proteins that can recognize effectors delivered into host cells by pathogens. The NBS domain is part of the NB-ARC domain that contains additional subdomains, including apoptotic protease-activating factor-1 (APAF-1), R gene products and caenorhabditis elegans death-4 protein (CED-4) (van der Biezen and Jones, 1998; Van Ooijen et al., 2008). NLR proteins are divided into two subclasses based on the N-terminal structure: TIR-NBS-LRR (TNL), which contains a toll-like-interleukin receptor (TIR) domain, and CC-NBS-LRR, which carries a coiled-coil (CC) domain (Han, 2019; Sun et al., 2020).

Five computational approaches have been developed for R protein prediction (Table 1). NLR-parser, RGAugury, and Restrepo-Montoya’s pipeline are alignment-based tools, and NBSPred and DRPPP are learning-based tools. NLR-parser uses motif alignment and search tool (MAST) to identify NLR-like sequences (Steuernagel et al., 2015). RGAugury identifies different subclasses of R proteins, including membrane-associated receptors (RLPs or RLKs) and NBS-containing proteins, by integrating the results generated from several computing programs, such as BLAST (Camacho et al., 2009), InterProScan (Zdobnov and Apweiler, 2001), HMMER3 (Eddy, 2011), nCoil (Lupas et al., 1991), and Phobius (Käll et al., 2004). Restrepo-Montoya et al. (2020) developed a computational approach to classify RLK and RLP proteins using SignalP 4.0 (Petersen et al., 2011), TMHMM 2 (Krogh et al., 2001) and PfamScan (Finn et al., 2014). However, methods based on sequence alignment are low-sensitive and time-consuming, which can lead to difficulties in predicting low similarity proteins. Machine learning-based methods, NBSPred and DRPPP, are used for the detection of R proteins based on SVM by considering various numerical representation schemes of protein sequences. NBSPred was developed to differentiate NLR/NLR-like proteins from non-NLR proteins. However, the NBSPred training datasets were generated by electronic searches and were not experimentally verified, which might reduce the accuracy of the model. DRPPP was built by extracting various features from input protein sequences, and the model achieved 91.11% accuracy for prediction plant R proteins. Unfortunately, the NBSPred^1^ and DRPPP^2^ web servers are no longer available.

**TABLE 1:** Summary of existing tools for plant R protein prediction.

Tool	Methods	Objects	Sites	References
NLR-parser	Motif alignment and search tool (MAST)	NLRs	http://github.com/steuernb/NLR-Parser	Steuernagel et al., 2015
RGAugury	BLAST search and domain/motif analysis	RLKs, RLPs, NLRs	https://bitbucket.org/yaanlpc/rgaugury	Li et al., 2016
Restrepo-Montoya’s method	BLAST search and domain/motif analysis	RLKs, RLPs	https://github.com/drestmont/plant_rlk_rlp/	Restrepo-Montoya et al., 2020
NBSPred	SVM	NLRs	http://soilecology.biol.lu.se/nbs/	Kushwaha et al., 2016
DRPPP	SVM	R proteins	http://14.139.240.55/NGS/download.php	Pal et al., 2016

*SVM, support vector machine.*

In this study, we developed an accurate computational approach for identifying R proteins using various sequence features. It is worth highlighting that the composition of k-spaced amino acid pairs (CKSAAPs) and k-spaced amino acid group pairs (CKSAAGPs) were also considered in the training process. The two-step feature selection strategy was adopted to detect irrelevant and redundant features. Then, the optimal *k* value and algorithm were evaluated for R protein prediction. Ultimately, support vector machine (SVM) and 5-spaced amino acid (group) pairs were chosen and applied to construct classifiers with sequence features.

## Materials and Methods

**FIGURE 1:**
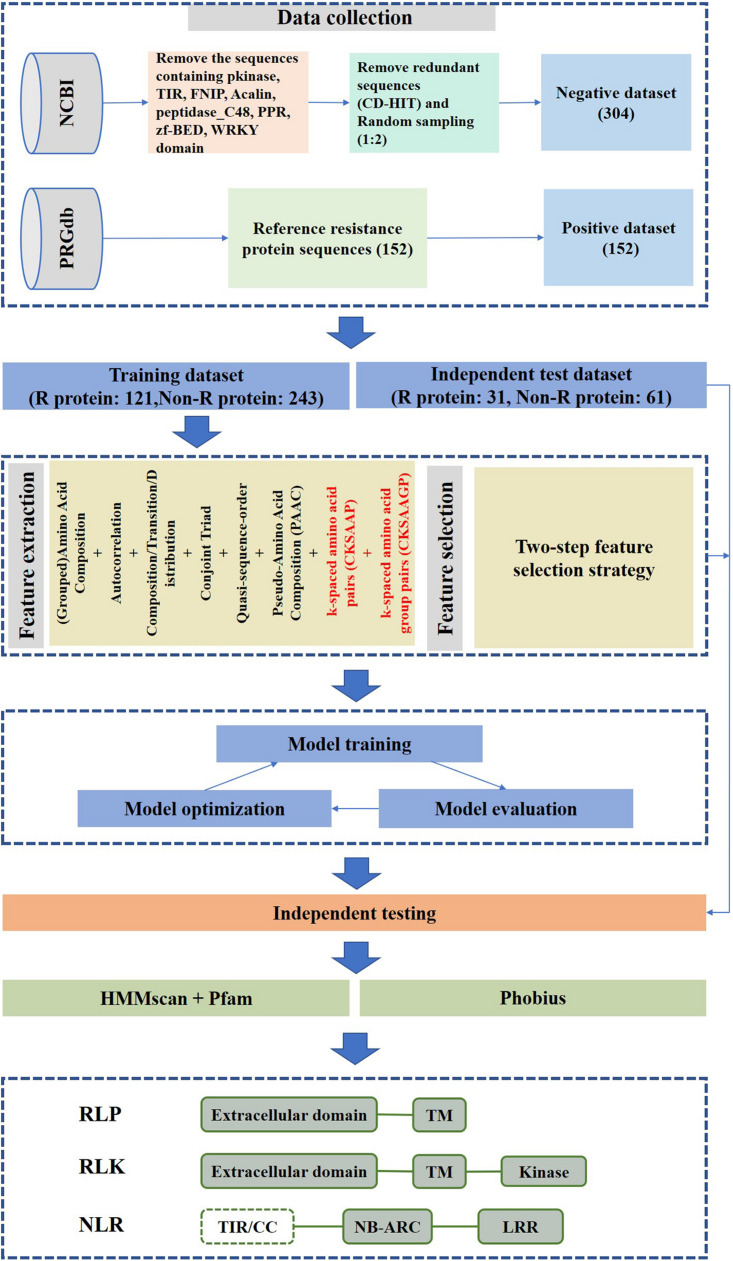
prPred workflow.

A flowchart of our method is shown in Figure 1. It can be summarized in five steps: (1) data collection; (2) feature construction; (3) two-step feature selection; (4) performance evaluation of features with or without CKSAAPs and CKSAAGPs; and (5) performance evaluation of different algorithms.

### Data Collection

We obtained plant R protein sequences from the PRGdb database^3^. R protein sequences were derived from 35 plant species and served as a positive dataset (Osuna-Cruz et al., 2018). Next, the known protein sequences of 35 plant species were downloaded from the NCBI protein database to construct a negative dataset. The sequences containing NB-ARC, LRR, Pkinase, TIR, FNIP, Acalin, peptidase_C48, PPR, zf-BED, and WRKY were filtered by a Pfam domain search (Kushwaha et al., 2016). To remove redundancy, proteins with sequence similarity >30% were excluded from the non-R protein dataset using CD-HIT (Fu et al., 2012). However, 34,975 protein sequences remained in the non-R protein dataset after filtering, thus, to ensure the balance of data, 304 protein sequences were selected randomly from the identified non-R proteins to serve as a final negative dataset. Then, 152 R proteins and 304 non-R proteins were split into training and test datasets at an 8:2 ratio. Finally, the training dataset is made up of 121 R protein sequences and 243 non-R protein sequences, and the independent test dataset is composed of 31 R protein sequences and 61 non-R protein sequences.

### Feature Construction

Features were extracted from input sequences using iFeature (Chen et al., 2018), such as amino acid composition, grouped amino acid composition, quasi-sequence-order, composition/transition/distribution (C/T/D), autocorrelation, conjoint triad and pseudo-amino acid composition (PseAAC). More detailed information about the features is described in the Supplementary Methods and Supplementary Table 1.

There are lots of feature extraction methods (Pal et al., 2016; Zeng et al., 2016; Liao et al., 2018; Zhang and Liu, 2019; Ikram et al., 2020; Li J. et al., 2020; Wang et al., 2020; Zhao et al., 2020; Zhu et al., 2020). In this work, we utilized CKSAAPs and CKSAAGPs as numeric vectors to represent the protein sequence. CKSAAP was used to calculate the occurrence frequencies of any two amino acids separated by any k amino acids. For example, if *k* = 0, the 0-spaced residue pairs can be represented as: AA, AC, AD, …, YY; if *k* = 1, the 1-spaced residue pairs can be expressed as AxA, AxC, AxD, …, YxY. The CKSAAPs are defined as:

$$k=0\left(\frac{\text{N}\left[\text{AA}\right]}{N_{0}},\frac{\text{N}\left[\text{AC}\right]}{N_{0}},\frac{\text{N}\left[\text{AD}\right]}{N_{0}},\ldots \ldots ,\frac{\text{N}\left[\text{YY}\right]}{N_{0}}\right)400$$

$$k=1\left(\frac{\text{N}\left[\text{AxA}\right]}{N_{1}},\frac{\text{N}\left[\text{AxC}\right]}{N_{1}},\frac{\text{N}\left[\text{AxD}\right]}{N_{1}},\ldots \ldots ,\frac{\text{N}\left[\text{YxY}\right]}{N_{1}}\right)400$$

$$k=2\left(\frac{\text{N}\left[\text{AxxA}\right]}{N_{2}},\frac{\text{N}\left[\text{AxxC}\right]}{N_{2}},\frac{\text{N}\left[\text{AxxD}\right]}{N_{2}},\ldots ,\frac{\text{N}\left[\text{YxxY}\right]}{N_{2}}\right)400$$

where “x” represents any of 20 amino acids; *N*_*k*_ was calculated as *N*_*k*_ = *L* − (*k* + 1), *k* = 1, 2, 3…, where *L* represents the length of a given protein sequence. The final feature vector was computed by concatenating the individual feature vectors; for example, if *k* = 5, the number of vector dimensions would be 400 × 6 = 2,400.

Amino acid residues can be divided into five categories based on chemical properties of the side chains, including aliphatic group (g1: GAVLMI), aromatic group (g2: FYW), positive charged group (g3: KRH), negative charged group (g4: DE), and uncharged group (g5: STCPNQ). k-spaced amino acid group pairs (CKSAAGP) is based on the frequency of two group separated by any k amino acids. If *k* = 0, the 0-spaced group pairs is represented as:

$$k=0\left(\frac{\text{N}\left[\text{g}1\text{g}1\right]}{N_{0}},\frac{\text{N}\left[\text{g}1\text{g}2\right]}{N_{0}},\frac{\text{N}\left[\text{g}1\text{g}3\right]}{N_{0}},\ldots \ldots ,\frac{\text{N}\left[\text{g}5\text{g}5\right]}{N_{0}}\right)25$$

### Two-Step Feature Selection Strategy

First, feature vectors were sorted according to the value of information gain (IG). A new feature list was generated in descending order of the IG value. Second, we selected or removed features based on the accuracy value during the training process. We added features from higher IG value to lower IG value. If the addition of a feature did not reduce the accuracy in the cross-validation strategy, then the feature vector was retained; otherwise, it was removed.

### Machine Learning Algorithms

Eight algorithms, including logistic regression (LR) (Hosmer et al., 2013), K-nearest neighbors (KNN) (Kramer, 2013), SVM (Hearst et al., 1998), decision tree (DT) (Swain and Hauska, 1977), random forest (RF) (Breiman, 2001), gradient boosting classifier (GBC) (Aler et al., 2017), Adaboost (Schapire, 2013), and extra-tree classifier (ETC) (Geurts et al., 2006), were chosen to train the model. We applied grid search (GS) to find optimal parameter combination in 10-fold cross-validation for each model. GS requires specifying a range for parameters, for example, the SVM parameter optimization using GS is implemented within the given ranges of *C* = {−5, 11} and γ = {−9, 13}.

### Performance Evaluation

To estimate the contributions of CKSAAPs and CKSAAGPs and to measure the overall predictive performance of the classification models, six parameters were applied for 10-fold cross-validation and independent tests (Hearst et al., 1998; An et al., 2019; Chen et al., 2019; Ding et al., 2019a, b; Fang et al., 2019; Jiang et al., 2019; Lv et al., 2019b, 2020b; Shen et al., 2019; Liu et al., 2020), including precision (Pre), sensitivity (Sen), specificity (Spe), accuracy (Acc), F1-score, and Matthew’s correlation coefficient (MCC). They are defined as follows:

(1) $$\text{Pre}=\frac{\text{TP}}{\text{TP}+\text{FP}}$$

(2) $$\text{Sen}=\frac{\text{TP}}{\text{TP}+\text{FN}}$$

(3) $$\text{Spe}=\frac{\text{TN}}{\text{FP}+\text{TN}}$$

(4) $$\text{Acc}=\frac{\text{TP}+\text{TN}}{\text{TP}+\text{FP}+\text{TN}+\text{FN}}$$

(5) $$\text{F}1-\text{score}=\frac{2\times \text{Pre}\times \text{Sen}}{\text{Pre}+\text{Sen}}$$

(6) $$\text{MCC}=\frac{\text{TP}\times \text{TN}-\text{FP}\times \text{FN}}{\sqrt{\left(\text{TP}+\text{FP}\right)\left(\text{TP}+\text{FN}\right)\left(\text{TN}+\text{FP}\right)\left(\text{TN}+\text{FN}\right)}}$$

where TP is the number of R proteins classified as R proteins, TN is the number of non-R proteins classified as non-R proteins, FP is the number of non-R proteins classified as R proteins, and FN is the number of R proteins classified as non-R proteins.

Additionally, the ROC curve and PR curve were used as visual assessment metrics. The ROC curve shows the false-positive rate versus the true positive rate, and the PR curve is recall versus precision. The area under the curve (AUC) is also provided as performance measure (Wang et al., 2010; Cheng et al., 2019). An AUC close to 1 indicates better prediction of the model.

## Results

### Comparison of Different Feature Combinations and Classification Models

CKSAAPs and CKSAAGPs are numerical encoding schemes that can capture short linear motif information, and the composition of CKSAAPs has been successfully applied to identify protein modification sites (Cheng et al., 2018; Lv et al., 2020c, d). We constructed feature vectors with CKSAAPs and CKSAAGPs because plant R proteins contain motif information distinct from that of non-R proteins (Supplementary Figure 1). The numerical encoding schemes of CKSAAP and CKSAAGP have exhibited obvious differences between R and non-R proteins using Wilcoxon rank sum test (Supplementary Figure 2). Table 2 showed that different models had different responses to the features with or without CKSAAPs and CKSAAGPs. For example, the Acc of LR showed no noticeable changes when CKSAAP and CKSAAGP features were added, while the Acc of SVM was improved from 0.902 to 0.935 in the independent dataset when considering 5-spaced amino acid pairs.

**TABLE 2:** Performance comparison of features with and without CKSAAP and CKSAAGP in the independent dataset test.

	Algorithms	Independent dataset test						
		Acc	Pre	Sen	Spe	F1-score	MCC	AUC
Without CKSAAPs and CKSAAGPs	LR	0.891	0.839	0.839	0.918	0.839	0.757	0.919
	KNN	0.891	0.862	0.806	0.934	0.833	0.754	0.928
	SVM	0.902	0.893	0.806	0.951	0.847	0.778	0.935
	RF	0.880	0.885	0.742	0.951	0.807	0.727	0.924
	DT	0.859	0.846	0.710	0.934	0.772	0.676	0.847
	GBC	0.815	0.733	0.710	0.869	0.721	0.583	0.839
	Adaboost	0.848	0.840	0.677	0.934	0.750	0.650	0.859
	ETC	0.913	0.926	0.806	0.967	0.862	0.803	0.947
*k* = 5	LR	0.891	0.862	0.806	0.934	0.833	0.754	0.946
	KNN	0.924	0.929	0.839	0.967	0.881	0.828	0.935
	SVM	**0.935**	**1.000**	**0.806**	**1.000**	**0.893**	**0.857**	**0.948**
	RF	0.913	0.960	0.774	0.984	0.857	0.805	0.931
	DT	0.880	0.917	0.710	0.967	0.800	0.729	0.854
	GBC	0.902	0.923	0.774	0.967	0.842	0.778	0.882
	Adaboost	0.870	0.828	0.774	0.918	0.800	0.704	0.880
	ETC	0.924	0.962	0.806	0.984	0.877	0.829	0.938

*LR, logistic regression; KNN, K nearest neighbors; SVM, support vector machine; RF, random forest; DT, decision tree; GBC, gradient boosting classifier; ETC, extra tree classifier. The bold values represent the predictive performanceof SVM based on 5-spaced amino acid pairs.*

**FIGURE 2:**
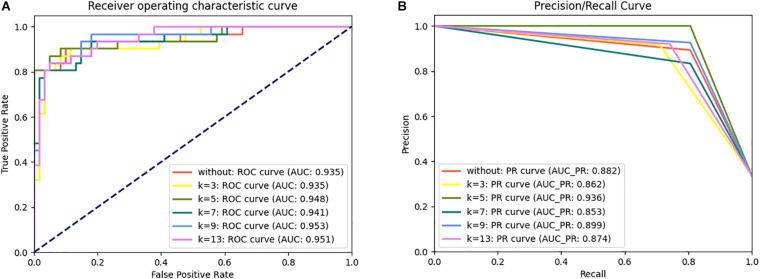
ROC **(A)** and PR **(B)** curve for the prPred classifier in the independent dataset test.

To determine the optimal algorithms and *k* value, we explored the discrimination power of *k* = 3-, 5-, 7-, 9-, and 13-spaced amino acid pairs using different algorithms (e.g., LR, KNN, SVM, RF, DT, GBC, Adaboost, and ETC) (Supplementary Table 2). We observed that SVM achieved better performance than other algorithms in 10-fold cross-validation tests in the same *k*-value. Although the AUC of SVM when *k* = 5 (AUC_*k* = 5_ = 0.948) was slightly lower than that when *k* = 9 and 13 (AUC_*k* = 9_ = 0.953, AUC_*k* = 13_ = 0.951) in the ROC curve in the independent dataset tests, the PR curve showed 4.12 and 7.09% improvements in AUC-PR when k = 5 compared with *k* = 9 and 13 (Figure 2). Moreover, the Acc, Spe, F1-score, and MCC values were improved by 2.41% (4.94%), 3.41% (3.41%), 3.60% (8.77%), and 6.72% (13.81%), respectively, compared with *k* = 9 (and 13) (Supplementary Table 2). Therefore, we chose SVM as the model and *k* = 5 to build the plant R protein predictor. The predictor showed satisfactory prediction results for the independent dataset with an Acc of 0.935, Pre of 1.000, Sen of 0.806, Spe of 1.000, F1-score of 0.893, MCC of 0.857, and AUC of 0.948 (Table 2 and Supplementary Table 2). The optimal parameters of SVM with the RBF kernel were *C* = 2.0 and γ = 0.0078.

### Prediction Pipeline of prPred

Because the published methods based on machine learning algorithms (e.g., NBSPred and DRPPP) are no longer available, performance comparisons cannot be carried out between prPred and the state-of-the-art methods. The alignment-based tools, NLR-parser and Restrepo-Montoya’s method are mainly applied to predict NLRs and PRRs (RLKs and RLPs), respectively. The RGAugury project aims to identify resistance gene analogs for plant genomes using interolog- and domain-based approaches. In the study, prPred integrated machine learning method and sequence alignment-based method to analyze and evaluate the potential R proteins. Except for predicting the potential R proteins, it was capable of annotating protein domain families based on Pfam-A using a hidden Markov model (HMM) and searching transmembrane regions (TMs) using Phobius to differentiate RLPs/PLKs from NLRs. Users can import protein sequences in FASTA format, and the prPred prediction results can be saved to CSV- and FASTA-formatted file. The CSV-formatted file output contains information about the protein sequence ID, prediction probability score, TM number, that as shown in Table 3.

**TABLE 3:** Example results in the CSV-format output file of prPred.

ID	R_protein_possibility	TM	SP	Domain				
Protein1	0.992151981	0	0	NB-ARC (PF00931.22)	Rx_N (PF18052.1)	LRR_8 (PF13855.6)	LRR_8 (PF13855.6)	LRR_8 (PF13855.6)
Protein2	0.992149469	0	0	NB-ARC (PF00931.22)	NB-ARC (PF00931.22)	Rx_N (PF18052.1)	Rx_N (PF18052.1)	Rx_N (PF18052.1)
Protein3	0.998599022	0	0	TIR (PF01582.20)	NB-ARC (PF00931.22)	NB-ARC (PF00931.22)	TIR_2 (PF13676.6)	
Protein4	0.992166647	1	Y	Pkinase (PF00069.25)	Pkinase_Tyr (PF07714.17)	LRRNT_2 (PF08263.12)	LRRNT_2 (PF08263.12)	LRR_8 (PF13855.6)
Protein5	0.992152188	1	Y	LRR_8 (PF13855.6)	LRR_8 (PF13855.6)	LRR_8 (PF13855.6)	LRR_8 (PF13855.6)	LRR_8 (PF13855.6)
Protein6	0.023914191	0	0					
Protein7	0.022744187	0	0	FHA (PF00498.26)				
Protein8	0.023851809	1	0					

## Conclusion

In this study, we developed a bioinformatics tool called prPred for the prediction of plant resistance proteins that combines CKSAAP and CKSAAGP features based on SVM. The predictive and analytical results demonstrated that the constructed model is an efficient predictor to distinguish R proteins from non-R proteins. CKSAAP and CKSAAGP features provide important improvements in the prediction performance. We expect that prPred will be a useful tool to facilitate biological research and provide guidance for related experimental validation. In the feature, we will use deep learning method and deep representation learning features for prPred (Lv et al., 2019a, 2020a, 2021; Li F. et al., 2020).

## Data Availability Statement

The datasets presented in this study can be found in online repositories. The names of the repository/repositories and accession number(s) can be found in the article/Supplementary Material.

## Author Contributions

YW and PW were responsible for experiments and manuscript preparation. YG and SH participated in discussions. YC and LX worked as supervisor for all procedures. All authors contributed to the article and approved the submitted version.

## Conflict of Interest

The authors declare that the research was conducted in the absence of any commercial or financial relationships that could be construed as a potential conflict of interest.

## Footnotes

1 http://soilecology.biol.lu.se/nbs/

2 http://14.139.240.55/NGS/download.php

3 http://prgdb.org/prgdb/
