# Supplementary material for: prPred: A Predictor to Identify Plant Resistance Proteins by Incorporating k-Spaced Amino Acid (Group) Pairs
Source: Front Bioeng Biotechnol. 2021 Jan 21;8:645520. doi: 10.3389/fbioe.2020.645520 (PMC7859348; doi:10.3389/fbioe.2020.645520)
Supplement: Supplementary file 1 [file Table_1.DOCX]

1. **Supplementary Method**

**Feature construction**

Amino acid composition descriptor covers the compositions of an individual amino acid, dipeptide and tripeptide in proteins. Amino acid residues can be divided into five categories based on chemical properties of the side chains, such as aliphatic group (g1: GAVLMI), aromatic group (g2: FYW), positive charged group (g3: KRH), negative charged group (g4: DE) and uncharged group (g5: STCPNQ). The grouped amino acid composition describes the frequency of each group in a given protein.

Pseudo amino acid composition (PseAAC) of a protein is represented by 20+λ discrete numbers in which the first 20 factors reflect amino acid composition information and the additional factors represent amino acid order information (Chou, 2001).

Quasi-sequence-order includes sequence-order-coupling number descriptors and quasi-sequence-order descriptors to reflect the possible sequence order patterns (Chou, 2000).

In C/T/D, C measures the frequency of 20 amino acids in the protein sequence. T represents the frequencies with which amino acids with special properties (e.g. hydrophobicity, hydrophobicity) is followed by amino acids with other properties. D stands for the distribution characteristics of the first, 25, 50, 75, and 100 percent of amino acids of the entire protein sequence (Dubchak et al., 1995).

Autocorrelation descriptor is based on the distribution of amino acid properties in sequence and measures the correlation between two residues in terms of their physicochemical properties. Three autocorrelation descriptors, including Moran, Geary, Normalized Moreau–Broto autocorrelation were calculated in this paper.

The conjoint triad considers the properties of one amino acid and its neighboring amino acids. It clusters the 20 amino acids into seven classes based on the dipoles and volumes of the side chains. The triad regards three continuous amino acids as a unit and if three amino acids belonging to the same class, they could be treated identically (Shen et al., 2007).

1. **Supplementary Tables**

**Table S1 Features were calculated in the study**

|  | Features |
| --- | --- |
| 1 | Amino acid composition (AAC) |
| 2 | Composition of k-spaced amino acid pairs (CKSAAP) |
| 3 | Dipeptide composition (DPC) |
| 4 | Dipeptide deviation from expected mean (DDE) |
| 5 | Tripeptide composition (TPC) |
| 6 | Grouped amino acid composition (GAAC) |
| 7 | Composition of k-spaced amino acid group pairs (CKSAAGP) |
| 8 | Grouped dipeptide composition (GDPC) |
| 9 | Grouped tripeptide composition (GTPC) |
| 10 | Moran (Moran) |
| 11 | Geary (Geary) |
| 12 | Normalized Moreau-Broto (NMBroto) |
| 13 | Composition (CTDC) |
| 14 | Transition (CTDT) |
| 15 | Distribution (CTDD) |
| 16 | Conjoint triad (CTriad) |
| 17 | Conjoint k-spaced triad (KSCTriad) |
| 18 | Sequence-order-coupling number (SOCNumber) |
| 19 | Quasi-sequence-order descriptors (QSOrder) |
| 20 | Pseudo-amino acid composition (PAAC) |
| 21 | Amphiphilic PAAC (APAAC) |

**Table S2 Performance of various classifiers and k values based on the 10-fold cross-validation test and independent dataset test**

|  | Algorithms | 10-fold cross-validation test | | | | |  | Independent dataset test | | | | | | |
| --- | --- | --- | --- | --- | --- | --- | --- | --- | --- | --- | --- | --- | --- | --- |
|  |  | Acc | Pre | Sen | F1-score | AUC |  | Acc | Pre | Sen | Spe | F1 | MCC | AUC |
| Without  CKSAAP and CKSAAGP | LR | 0.904±0.051 | 0.864±0.088 | 0.851±0.117 | 0.852±0.084 | 0.959±0.029 |  | 0.891 | 0.839 | 0.839 | 0.918 | 0.839 | 0.757 | 0.919 |
|  | KNN | 0.907±0.053 | 0.923±0.099 | 0.794±0.104 | 0.849±0.083 | 0.945±0.037 |  | 0.891 | 0.862 | 0.806 | 0.934 | 0.833 | 0.754 | 0.928 |
|  | SVM | 0.915±0.043 | 0.916±0.072 | 0.827±0.125 | 0.862±0.078 | 0.961±0.039 |  | 0.902 | 0.893 | 0.806 | 0.951 | 0.847 | 0.778 | 0.935 |
|  | RF | 0.923±0.036 | 0.970±0.046 | 0.794±0.090 | 0.871±0.063 | 0.952±0.050 |  | 0.880 | 0.885 | 0.742 | 0.951 | 0.807 | 0.727 | 0.924 |
|  | DT | 0.887±0.038 | 0.961±0.063 | 0.694±0.112 | 0.799±0.077 | 0.899±0.048 |  | 0.859 | 0.846 | 0.710 | 0.934 | 0.772 | 0.676 | 0.847 |
|  | GBC | 0.884±0.040 | 0.854±0.066 | 0.792±0.114 | 0.817±0.072 | 0.936±0.064 |  | 0.815 | 0.733 | 0.710 | 0.869 | 0.721 | 0.583 | 0.839 |
|  | Adaboost | 0.849±0.050 | 0.755±0.080 | 0.819±0.096 | 0.782±0.073 | 0.880±0.062 |  | 0.848 | 0.840 | 0.677 | 0.934 | 0.750 | 0.650 | 0.859 |
|  | ETC | 0.915±0.043 | 0.949±0.051 | 0.786±0.111 | 0.856±0.079 | 0.961±0.045 |  | 0.913 | 0.926 | 0.806 | 0.967 | 0.862 | 0.803 | 0.947 |
| k=3 | LR | 0.915±0.048 | 0.878±0.083 | 0.868±0.100 | 0.870±0.077 | 0.958±0.036 |  | 0.902 | 0.893 | 0.806 | 0.951 | 0.847 | 0.778 | 0.915 |
|  | KNN | 0.906±0.047 | 0.936±0.078 | 0.778±0.116 | 0.844±0.079 | 0.949±0.032 |  | 0.902 | 0.893 | 0.806 | 0.951 | 0.847 | 0.778 | 0.919 |
|  | SVM | 0.926±0.040 | 0.969±0.048 | 0.802±0.106 | 0.874±0.076 | 0.960±0.040 |  | 0.880 | 0.917 | 0.710 | 0.967 | 0.800 | 0.729 | 0.935 |
|  | RF | 0.918±0.044 | 0.968±0.050 | 0.778±0.110 | 0.859±0.081 | 0.948±0.047 |  | 0.902 | 0.923 | 0.774 | 0.967 | 0.842 | 0.778 | 0.925 |
|  | DT | 0.871±0.064 | 0.901±0.082 | 0.678±0.151 | 0.769±0.125 | 0.822±0.086 |  | 0.815 | 0.792 | 0.613 | 0.918 | 0.691 | 0.572 | 0.765 |
|  | GBC | 0.909±0.042 | 0.921±0.074 | 0.803±0.116 | 0.852±0.075 | 0.935±0.062 |  | 0.870 | 0.880 | 0.710 | 0.951 | 0.786 | 0.702 | 0.903 |
|  | Adaboost | 0.890±0.056 | 0.812±0.105 | 0.894±0.089 | 0.846±0.076 | 0.909±0.061 |  | 0.826 | 0.857 | 0.581 | 0.951 | 0.692 | 0.599 | 0.818 |
|  | ETC | 0.923±0.042 | 0.959±0.051 | 0.802±0.106 | 0.871±0.078 | 0.963±0.045 |  | 0.913 | 0.926 | 0.806 | 0.967 | 0.862 | 0.803 | 0.946 |
| k=5 | LR | 0.896±0.042 | 0.849±0.074 | 0.844±0.100 | 0.842±0.066 | 0.955±0.029 |  | 0.891 | 0.862 | 0.806 | 0.934 | 0.833 | 0.754 | 0.946 |
|  | KNN | 0.890±0.048 | 0.870±0.083 | 0.794±0.104 | 0.827±0.078 | 0.941±0.039 |  | 0.924 | 0.929 | 0.839 | 0.967 | 0.881 | 0.828 | 0.935 |
|  | SVM | 0.923±0.040 | 0.959±0.051 | 0.802±0.099 | 0.871±0.074 | 0.954±0.047 |  | **0.935** | **1.000** | **0.806** | **1.000** | **0.893** | **0.857** | **0.948** |
|  | RF | 0.920±0.043 | 0.961±0.064 | 0.794±0.106 | 0.866±0.079 | 0.963±0.039 |  | 0.913 | 0.960 | 0.774 | 0.984 | 0.857 | 0.805 | 0.931 |
|  | DT | 0.879±0.035 | 0.938±0.052 | 0.687±0.114 | 0.786±0.072 | 0.853±0.070 |  | 0.880 | 0.917 | 0.710 | 0.967 | 0.800 | 0.729 | 0.854 |
|  | GBC | 0.884±0.046 | 0.878±0.086 | 0.769±0.121 | 0.813±0.079 | 0.932±0.030 |  | 0.902 | 0.923 | 0.774 | 0.967 | 0.842 | 0.778 | 0.882 |
|  | Adaboost | 0.860±0.064 | 0.775±0.104 | 0.826±0.115 | 0.796±0.097 | 0.893±0.058 |  | 0.870 | 0.828 | 0.774 | 0.918 | 0.800 | 0.704 | 0.880 |
|  | ETC | 0.918±0.045 | 0.944±0.072 | 0.803±0.110 | 0.863±0.081 | 0.955±0.036 |  | 0.924 | 0.962 | 0.806 | 0.984 | 0.877 | 0.829 | 0.938 |
| k=7 | LR | 0.921±0.054 | 0.881±0.096 | 0.885±0.106 | 0.880±0.085 | 0.963±0.037 |  | 0.902 | 0.893 | 0.806 | 0.951 | 0.847 | 0.778 | 0.928 |
|  | KNN | 0.909±0.039 | 0.937±0.069 | 0.786±0.104 | 0.850±0.068 | 0.959±0.034 |  | 0.891 | 0.889 | 0.774 | 0.951 | 0.828 | 0.753 | 0.920 |
|  | SVM | 0.932±0.052 | 0.929±0.075 | 0.860±0.118 | 0.889±0.090 | 0.966±0.034 |  | 0.880 | 0.833 | 0.806 | 0.918 | 0.820 | 0.730 | 0.941 |
|  | RF | 0.912±0.047 | 0.925±0.082 | 0.802±0.084 | 0.858±0.077 | 0.949±0.051 |  | 0.870 | 0.852 | 0.742 | 0.934 | 0.793 | 0.702 | 0.930 |
|  | DT | 0.862±0.045 | 0.811±0.113 | 0.801±0.107 | 0.795±0.056 | 0.859±0.046 |  | 0.848 | 0.758 | 0.806 | 0.869 | 0.781 | 0.666 | 0.852 |
|  | GBC | 0.890±0.056 | 0.887±0.091 | 0.770±0.118 | 0.821±0.095 | 0.941±0.057 |  | 0.859 | 0.846 | 0.710 | 0.934 | 0.772 | 0.676 | 0.885 |
|  | Adaboost | 0.876±0.060 | 0.796±0.100 | 0.860±0.104 | 0.823±0.084 | 0.899±0.065 |  | 0.837 | 0.864 | 0.613 | 0.951 | 0.717 | 0.625 | 0.807 |
|  | ETC | 0.923±0.042 | 0.968±0.049 | 0.794±0.106 | 0.869±0.078 | 0.969±0.036 |  | 0.913 | 0.926 | 0.806 | 0.967 | 0.862 | 0.803 | 0.936 |
| k=9 | LR | 0.915±0.048 | 0.880±0.084 | 0.868±0.119 | 0.869±0.083 | 0.957±0.038 |  | 0.891 | 0.862 | 0.806 | 0.934 | 0.833 | 0.754 | 0.937 |
|  | KNN | 0.904±0.052 | 0.918±0.094 | 0.786±0.117 | 0.842±0.089 | 0.960±0.033 |  | 0.891 | 0.920 | 0.742 | 0.967 | 0.821 | 0.753 | 0.933 |
|  | SVM | 0.932±0.056 | 0.942±0.079 | 0.843±0.120 | 0.887±0.097 | 0.967±0.034 |  | **0.913** | **0.926** | **0.806** | **0.967** | **0.862** | **0.803** | **0.953** |
|  | RF | 0.923±0.042 | 0.968±0.050 | 0.794±0.100 | 0.870±0.077 | 0.958±0.044 |  | 0.880 | 0.885 | 0.742 | 0.951 | 0.807 | 0.727 | 0.943 |
|  | DT | 0.852±0.067 | 0.787±0.121 | 0.785±0.100 | 0.781±0.092 | 0.844±0.055 |  | 0.859 | 0.800 | 0.774 | 0.902 | 0.787 | 0.681 | 0.838 |
|  | GBC | 0.898±0.050 | 0.891±0.072 | 0.794±0.113 | 0.836±0.086 | 0.942±0.045 |  | 0.880 | 0.885 | 0.742 | 0.951 | 0.807 | 0.727 | 0.919 |
|  | Adaboost | 0.887±0.053 | 0.831±0.119 | 0.860±0.081 | 0.838±0.069 | 0.901±0.044 |  | 0.826 | 0.857 | 0.581 | 0.951 | 0.692 | 0.599 | 0.821 |
|  | ETC | 0.920±0.041 | 0.959±0.051 | 0.794±0.106 | 0.865±0.077 | 0.969±0.033 |  | 0.913 | 0.926 | 0.806 | 0.967 | 0.862 | 0.803 | 0.947 |
| k=13 | LR | 0.912±0.058 | 0.867±0.095 | 0.876±0.125 | 0.867±0.095 | 0.969±0.027 |  | 0.902 | 0.893 | 0.806 | 0.951 | 0.847 | 0.778 | 0.940 |
|  | KNN | 0.912±0.036 | 0.939±0.066 | 0.794±0.111 | 0.854±0.066 | 0.964±0.029 |  | 0.891 | 0.862 | 0.806 | 0.934 | 0.833 | 0.754 | 0.936 |
|  | SVM | 0.934±0.042 | 0.988±0.038 | 0.810±0.111 | 0.887±0.080 | 0.968±0.035 |  | **0.891** | **0.920** | **0.742** | **0.967** | **0.821** | **0.753** | **0.951** |
|  | RF | 0.923±0.042 | 0.960±0.050 | 0.802±0.112 | 0.870±0.078 | 0.958±0.046 |  | 0.891 | 0.862 | 0.806 | 0.934 | 0.833 | 0.754 | 0.884 |
|  | DT | 0.876±0.051 | 0.883±0.096 | 0.727±0.119 | 0.792±0.094 | 0.839±0.066 |  | 0.837 | 0.833 | 0.645 | 0.934 | 0.727 | 0.624 | 0.790 |
|  | GBC | 0.901±0.050 | 0.906±0.082 | 0.785±0.100 | 0.839±0.084 | 0.929±0.068 |  | 0.859 | 0.821 | 0.742 | 0.918 | 0.780 | 0.678 | 0.901 |
|  | Adaboost | 0.885±0.038 | 0.794±0.071 | 0.894±0.089 | 0.837±0.056 | 0.914±0.049 |  | 0.826 | 0.857 | 0.581 | 0.951 | 0.692 | 0.599 | 0.804 |
|  | ETC | 0.920±0.041 | 0.959±0.051 | 0.794±0.106 | 0.865±0.077 | 0.964±0.034 |  | 0.913 | 0.926 | 0.806 | 0.967 | 0.862 | 0.803 | 0.953 |

LR, Logistic Regression; KNN, K Nearest Neighbors; SVM, Support Vector Machine; RF, Random Forest; DT, Decision Tree; GBC, Gradient Boosting Classifier; ETC, Extra Tree Classifier

1. **Supplementary Figures**


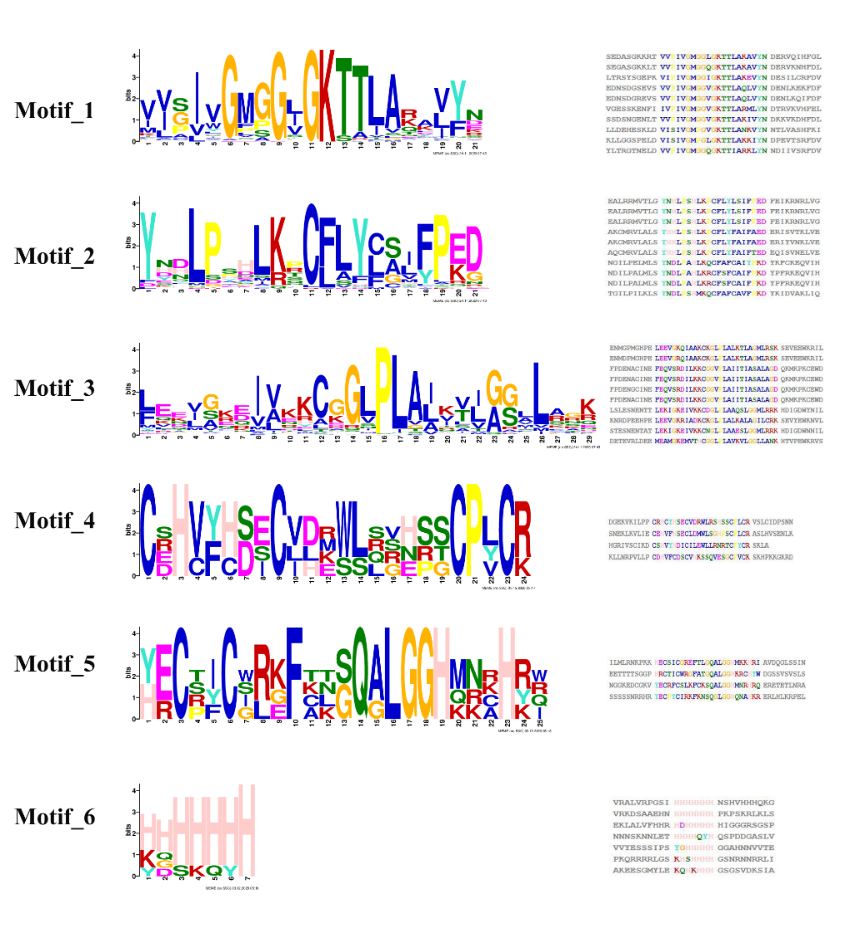


**Figure S1 Motifs in plant R (motif 1-3) and non-R (motif 4-6) proteins**


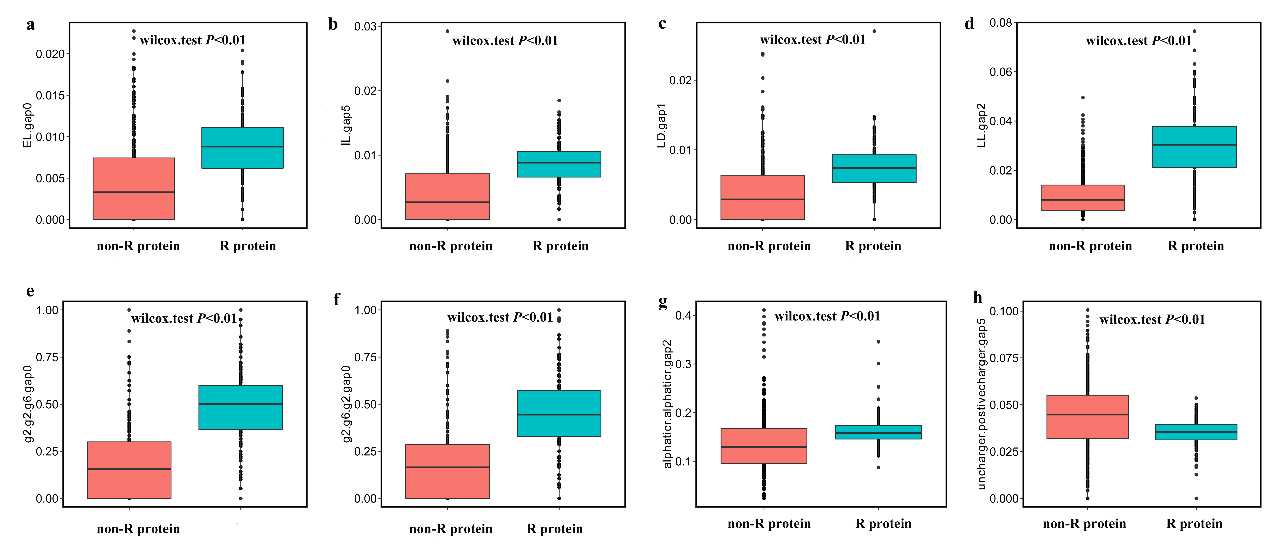


**Figure S2** **Significant differences of CKSAAP (a-d) and CKSAAGP (e-h) between R and non-R proteins**

**References**

Chou, K.-C. (2000). Prediction of protein subcellular locations by incorporating quasi-sequence-order effect. *Biochem. Bioph. Res. Co.* 278, 477-483. doi: 410.1006/bbrc.2000.3815.

Chou, K.C. (2001). Prediction of protein cellular attributes using pseudo‐amino acid composition. *Proteins: Structure, Function, and Bioinformatics* 43, 246-255. doi: 210.1002/prot.1035.

Dubchak, I., Muchnik, I., Holbrook, S.R., et al. (1995). Prediction of protein folding class using global description of amino acid sequence. *P. Natl. Acad. Sci. USA* 92, 8700-8704. doi: 8710.1073/pnas.8792.8719.8700.

Shen, J., Zhang, J., Luo, X., et al. (2007). Predicting protein–protein interactions based only on sequences information. *P. Natl. Acad. Sci. USA* 104, 4337-4341. doi: 4310.1073/pnas.0607879104.
